# Patents

**Published:** 1874-11

**Authors:** 


### PATENTS.

FOR years the present proprietors of HALL'S JOURNAL OF HEALTH were largely engaged in conducting business before the United States Patent Office. Their duties to the JOURNAL have rendered it impossible for them to devote time to this branch of business, and they have been compelled to abandon it, so far as personal attention is concerned. But, as many of their old customers still apply to them, they here take occasion to say that they still receive models and descriptions of new inventions, which they at once place in the hands of a first-class solicitor, who faithfully attends to the work, and to whom they give, if needed, the benefit of their advice. Inventors can, therefore, as heretofore, send all such matters to the publishers of HALL'S JOURNAL OF HEALTH, 84 Broadway, and be sure of intelligent and honest assistance.

